# Within-Host Speciation of Malaria Parasites

**DOI:** 10.1371/journal.pone.0000235

**Published:** 2007-02-21

**Authors:** Javier Pérez-Tris, Olof Hellgren, Asta Križanauskienė, Jonas Waldenström, Jean Secondi, Camille Bonneaud, Jon Fjeldså, Dennis Hasselquist, Staffan Bensch

**Affiliations:** 1 Department of Animal Ecology, Lund University, Lund, Sweden; 2 Department of Zoology and Physical Anthropology, Complutense University, Madrid, Spain; 3 Institute of Ecology, Vilnius University, Vilnius, Lithuania; 4 Laboratoire d'Ecologie Animale, Université d'Angers, Angers, France; 5 Laboratoire de Parasitologie Evolutive, Université Pierre et Marie Curie, Paris, France; 6 Zoological Museum, University of Copenhagen, Copenhagen, Denmark; Oxford University, United Kingdom

## Abstract

**Background:**

Sympatric speciation—the divergence of populations into new species in absence of geographic barriers to hybridization—is the most debated mode of diversification of life forms. Parasitic organisms are prominent models for sympatric speciation, because they may colonise new hosts within the same geographic area and diverge through host specialization. However, it has been argued that this mode of parasite divergence is not strict sympatric speciation, because host shifts likely cause the sudden effective isolation of parasites, particularly if these are transmitted by vectors and therefore cannot select their hosts. Strict sympatric speciation would involve parasite lineages diverging within a single host species, without any population subdivision.

**Methodology/Principal Findings:**

Here we report a case of extraordinary divergence of sympatric, ecologically distinct, and reproductively isolated malaria parasites within a single avian host species, which apparently occurred without historical or extant subdivision of parasite or host populations.

**Conclusions/Significance:**

This discovery of within-host speciation changes our current view on the diversification potential of malaria parasites, because neither geographic isolation of host populations nor colonization of new host species are any longer necessary conditions to the formation of new parasite species.

## Introduction

Malaria parasites comprise a diverse group of protozoans that infect reptiles, mammals and birds, and that are transmitted through the bite of different families of blood-feeding insect vectors [1], [2]. They encompass two closely related genera, *Plasmodium* and *Haemoproteus* [2], which contain about 170 and 150 morphologically distinct species, respectively [1], and many more cryptic species as revealed by DNA sequencing of mitochondrial and nuclear gene fragments [3]. Genetic techniques have substantially improved our understanding of both the diversity and the modes of lineage divergence in non-human malaria parasites [4]. Speciation in malaria parasites is now known to follow from the subdivision of parasite populations in discrete habitats, such as different geographic regions in which host populations become isolated, or different host species accidentally colonised with the assistance of vectors [4], [5]. Such findings fit to the expectations of usual modes of allopatric and sympatric speciation, respectively, which have been proposed for parasitic organisms [6]–[9]. In contrast, theoretical expectations of strict sympatric, within-host speciation involve the evolution of reproductive isolation among the members of an interbreeding population of parasites within a single host species [9]. Such a mode of diversification can be inferred from phylogenetic relationships and host distributions of extant parasite species, the critical observation being a fully sympatric community of reproductively isolated sister parasite lineages within a geographically unstructured host species [8], [9].

Here we describe a case of great diversification of *Haemoproteus* parasite lineages that has occurred within a single bird host species, the blackcap *Sylvia atricapilla*. To illustrate such occurrence, we (i) determine the distribution of parasite lineages among 47 passerine species that are sympatric to the blackcap, showing that most blackcap parasites are exclusive to this species, (ii) determine the evolutionary relationships of parasites found in blackcaps and its closest relatives, demonstrating that many blackcap parasites are included in a monophyletic group that apparently diverged within the blackcap, (iii) analyse whether such group of parasites are reproductively isolated entities, and (iv) analyse the geographic structuring of genetic variation of blackcap parasites, showing that the probability that this group of parasites evolved in allopatry is very slim.

## Results

### Parasite screening

We sequenced part of the mitochondrial cytochrome *b* gene of malaria parasites found in European passerine birds to broadly investigate the phylogenetic diversity of this group of organisms. Our survey included 4470 individual birds of 47 European species, and 1911 parasite infections, each one involving one to four parasite lineages distinguished through their DNA sequences [3]. We were able to identify whether parasites belonged to the *Plasmodium* (45 lineages) or the *Haemoproteus* (92 lineages) genera by comparing their DNA sequences with those of parasites identified by microscopy.

### Parasite richness in the blackcap

**Figure 1:**
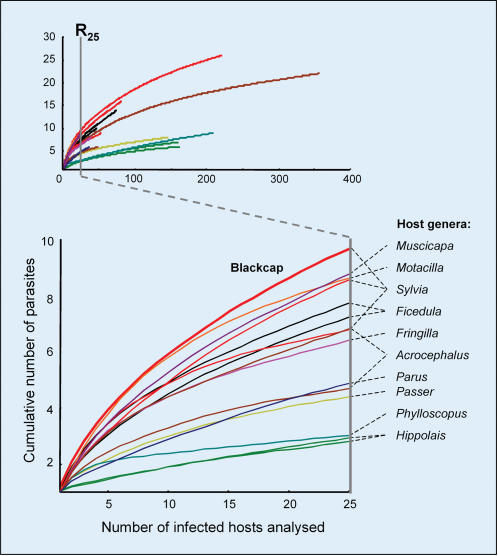
Variation in richness of malaria parasites among different bird host species. The curves represent parasite cumulative richness in 15 passerine species with more than 25 scored parasite infections, belonging to 10 bird genera (represented in different colours, see details in Table 1). The curves provide a standard estimate of parasite richness (R_25_) for each species, given by the number of parasites accumulated after analysing 25 infected individuals. The small graph shows the curves of parasite accumulation including all infections scored in each species (n = 26-353), crossed by a dotted line at R_25_. Blackcaps show the highest richness of malaria parasites in both graphs.

One warbler species, the blackcap, was exceptionally rich in parasites, harbouring 19% of all parasites found among the 47 screened European bird species. This pattern remained when we restricted the analysis to widely sampled bird species, for which we had scored at least 25 parasite infections. For these species, we could estimate intraspecific parasite richness independent of sampling effort as the number of parasite lineages accumulated in 25 scored infections (an index we named R_25_). This index of parasite richness was higher in blackcaps (R_25_ = 9.6±0.06) than in any other widely sampled bird species (14 species with more than 25 scored infections: R_25_ = 6.0±0.58, Fig. 1). Most importantly, blackcaps had the largest proportion of exclusive parasites observed in any species studied (73.1%), with other widely sampled birds harbouring a significantly lower number of exclusive lineages (33.1%±4.8, higher 95% confidence limit  = 68.4%, *P*<0.05, Table 1). Sampling bias did not explain these results, as blackcaps showed higher parasite diversity than other bird species for which the sampling effort was often higher (Fig. 1).

**Table 1:** Sampling details of the 48 bird species included in this study (part one).

	Sample size		Parasite richness		Exclusive lineages	
Host species	Individuals	Infections	*n* lineages	R_25_	*n* exclusive	% exclusive
*Acrocephalus arundinaceus*	858	357	22	6.8 ± 0.05	7	31.8
*Acrocephalus palustris*	30	17	7		0	
*Acrocephalus scirpaceus*	69	20	10		1	
*Acrocephalus schoenobaenus*	122	50	6	4.7 ± 0.03	0	0
*Anthus trivialis*	7	2	2		1	
*Carduelis spinus*	7	6	1		0	
*Carpodacus erythrinus*	5	5	3		1	
*Cercotrichas galactotes*	6	3	3		1	
*Coccothraustes coccothraustes*	6	6	4		0	
*Emberiza schoeniclus*	7	1	1		0	
*Erithacus rubecula*	4	3	2		0	
*Ficedula albicollis*	236	74	14	7.8 ± 0.05	3	21.4
*Ficedula hypoleuca*	81	47	10	7.3 ± 0.04	0	0
*Fringilla coelebs*	51	40	8	6.4 ± 0.03	5	62.5
*Fringilla montifringilla*	9	4	2		1	
*Hippolais icterina*	192	162	7	2.9 ± 0.03	4	57.1
*Hippolais pallida*	32	8	4		3	
*Hippolais polyglotta*	183	164	6	2.8 ± 0.03	2	33.3
*Hirundo rustica*	1	1	1		1	
*Lanius collurio*	83	14	7		4	
*Loxia curvirostra*	17	4	1		0	
*Luscinia luscinia*	7	6	5		1	
*Luscinia megarhynchos*	35	16	4		3	
*Luscinia svecica*	86	21	9		1	
*Motacilla alba*	1	1	1		1	
*Motacilla flava*	144	26	9	8.6 ± 0.02	4	44.4
*Muscicapa striata*	41	26	9	8.8 ± 0.01	4	44.4
*Oenanthe oenanthe*	31	3	2		0	
*Parus ater*	3	2	3		0	
*Parus caeruleus*	17	15	2		0	
*Parus major*	42	37	6	4.9 ± 0.03	2	33.3
*Parus montanus*	2	2	1		0	
*Parus palustris*	4	3	1		0	
*Passer domesticus*	232	146	8	4.4 ± 0.03	2	25.0
*Phoenicurus phoenicurus*	46	9	4		1	
*Phylloscopus collybita*	1	1	1		0	
*Phylloscopus sibillatrix*	3	3	1		0	
*Phylloscopus trochilus*	943	209	9	3.0 ± 0.03	3	33.3
*Saxicola rubetra*	20	9	5		0	
*Sylvia (Pseudoalcippe) abyssinica*	43	16	7		6	
***Sylvia atricapilla***	**415**	**222**	**26**	**9.6 ± 0.06**	**19**	**73.1**
*Sylvia borin*	275	83	16	8.6 ± 0.05	7	43.8
*Sylvia communis*	77	54	9	6.8 ± 0.03	3	33.3
*Sylvia curruca*	12	10	6		2	
*Sylvia melanocephala*	12	5	1		0	
*Sylvia nisoria*	2	2	2		1	
*Turdus merula*	12	11	2		0	
*Turdus philomelos*	2	2	2		1	
Total	4513	1927				

For each bird species, the table shows the number of individuals screened and the number of infections scored. Parasite richness is given both as a raw value for all species, and also as R25 (± S.E.) for 15 species with more than 25 scored infections. The percentage of exclusive parasite lineages is only calculated for widely sampled bird species (with more than 25 scored infections). The blackcap entries have been boldfaced.

### Endemism of blackcap parasites

**Figure 2:**
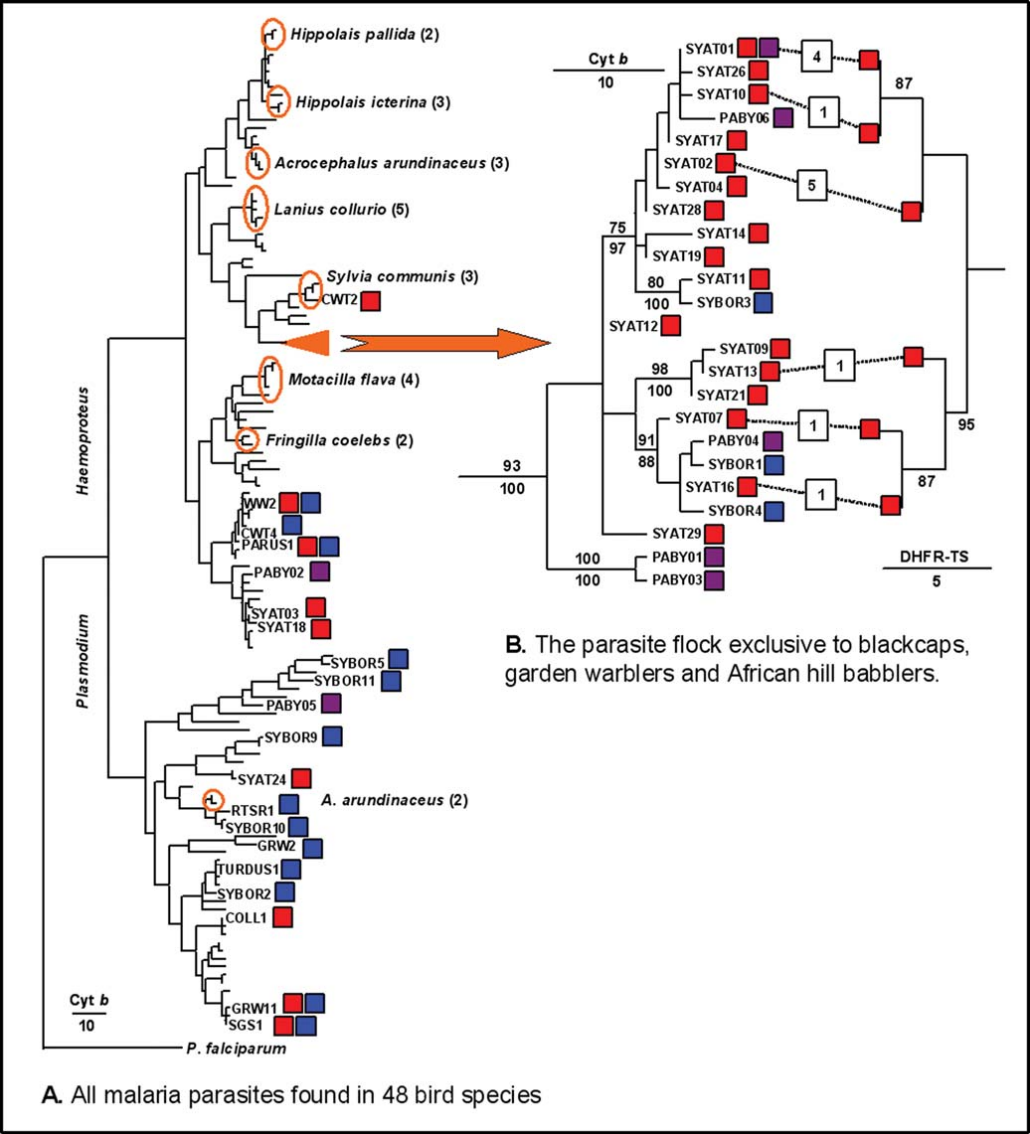
The blackcap parasite flock placed in a phylogenetic context. The tree A shows the phylogenetic position of parasites found in blackcaps (red), garden warblers (blue) and African hill babblers (purple), among 143 parasite lineages found in 48 bird species. Monophyletic parasite groups apparently exclusive to some species are encircled in orange (species names are mentioned and the number of parasites in each group is shown in brackets). The tree B shows the parasite flock (represented by a triangle in the tree A). Numbers represent >75% support to internal branches, based on bootstrap replicates (above) or posterior probabilities (below). For six parasites in this cluster, the figure shows the match between phylogenies based on mitochondrial cytochrome *b* and nuclear DHFR-TS genes (the number of cases with each association of haplotypes is indicated, and bootstrap support is reported if >75%).

Seventeen parasites found only in blackcaps, summing up 18.5% of all *Haemoproteus* parasites found in 47 European host species, made the bulk of a monophyletic group in the genus *Haemoproteus* (Fig. 2). The microscopic investigation of various parasites from this group places them within the morphological species *H. parabelopolskyi* [10]. These blackcap parasites are not shared with African birds species either, according to an extensive survey (over 5800 individuals) of European and African birds, including more than 100 African passerine species [11], which strongly supports endemism of this group of parasites in blackcaps. A search in GenBank (run in January 23, 2007) failed to find any parasite sequence within this monophyletic group that had been retrieved from any bird species other than the blackcap. However, the search identified some of the sequences found in our study as blackcap parasites independently found in places not included in our survey. Given that GenBank includes hundreds of cytochrome *b* sequences of *Haemoproteus* parasites retrieved from hundreds of bird species sampled worldwide, the result of our search further supports endemism of blackcap parasites.

**Figure 3:**
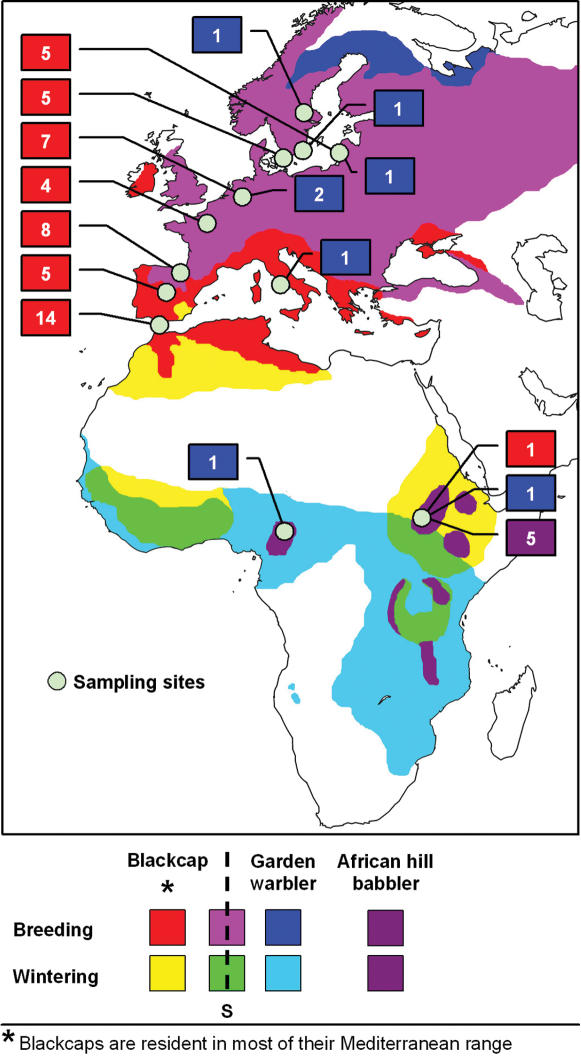
The geographic distribution of the parasite flock. The map shows the location of our sampling sites within the range of blackcaps, garden warblers and African hill babblers, which are shadowed in different colours according to the key below the map. Blackcaps and garden warblers are sympatric (S) in wide areas of Europe and Africa, during the breeding season and in winter, respectively. African hill babblers are year-round residents. Many blackcaps and garden warblers from Europe spend the winter in the range of African hill babblers, where the three species occur in the same habitat. For each sampling site, the squares indicate the number of parasite lineages from the flock that were found in each species (blackcap parasites in red, garden warbler parasites in blue, and hill babbler parasites in purple).

This phylogenetic “flock of parasites” also included three parasites infecting only garden warblers *Sylvia borin*, the closest extant relative species to the blackcap. However, garden warbler parasites were never found infecting blackcaps, or vice versa (*n* = 179 blackcaps and 54 garden warblers infected by parasites of this group; Fisher exact *P*<0.0001), despite of the fact that both species are sympatric over most of their ranges and were often trapped at the same sites (Fig. 3), supporting high host specificity in this group of closely related parasites. Within our sample, other bird species often harboured nearly as many parasites as the blackcap (Table 1). However, the blackcap was unique in that the majority of its parasites were sister lineages not found in other species. Other species never had more than five parasites forming monophyletic groups, even if the parasites of several closely related bird species were considered together (Fig. 2).

Given that blackcaps harboured many more of these monophyletic parasites than garden warblers, we suspected that parasites might have diversified in blackcaps after both bird species diverged from each other, and then some parasite lineages switched host from blackcaps to garden warblers (Fig. 2). To further examine this possibility, we analysed a sample of African hill babblers (*Sylvia [Pseudoalcippe] abyssinica*), which is the closest extant relative to the species pair formed by blackcaps and garden warblers [12]. Among 43 screened hill babblers, we found 16 infections involving 7 parasite lineages. Three of these parasites were included in the monophyletic group infecting blackcaps and garden warblers (one of them shared with blackcaps), and another two formed an outgroup to the above parasite cluster, supporting the possibility that parasites cospeciated with their host species when blackcaps and garden warblers diverged from African hill babblers (Fig. 2). We obtained good statistical support for the monophyly of the parasite group shared by all three species (93–100%), as well as for the early divergence of two African hill babbler parasites in the evolutionary history of the group (100%), despite the minor mtDNA sequence differences among these parasites and their closest relatives (Fig. 2). Because of the latter reason, however, the evolutionary relationships between the three host species and the parasites in the flock were difficult to establish. Frequent host switching might drive parasite divergence in this system, but such scenario is unlikely because it implies newly diverged parasites nearly always switching host to blackcaps and becoming extinct in the other two host species, although the three host species are sympatric. In our view, the three bird species likely acquired their distinct parasites from a common ancestor, and later on such parasites went through substantial diversification in blackcaps, dramatically more than in garden warblers or in African hill babblers. At this point, we do not have a plausible explanation that can explain why parasite diversification may have been so much stronger in the blackcap than in the other investigated passerines.

### Reproductive isolation of blackcap parasites

**Figure 4:**
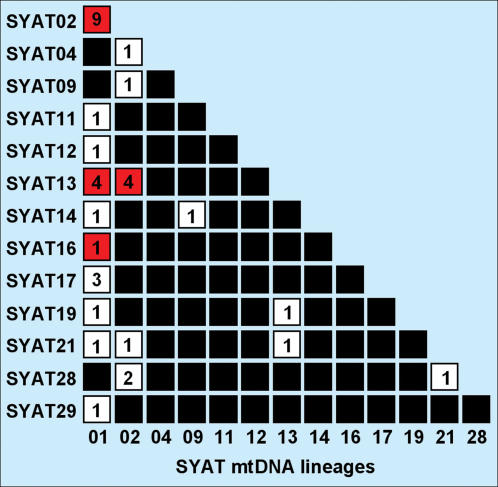
Co-occurrence of sister blackcap parasites (see tree in Fig. 2B) in mixed infections of individual hosts. Numbers indicate the frequency of occurrence of each parasite combination. Combinations in red involve parasites for which frequent hybridisation is unlikely, according to linkage between mitochondrial and nuclear haplotypes.

To evaluate whether sister blackcap parasites are reproductively isolated, we sequenced part of the nuclear DHFR-TS gene of parasites in 13 infections involving six members of the aforementioned *Haemoproteus* parasite flock. Both cytochrome *b* and DHFR-TS genes produced identical phylogenies, although two mitochondrial lineages shared identical nuclear sequences (Fig. 2). The latter case might signify intraspecific polymorphism, or conservation of the nuclear sequence between reproductively isolated parasite lineages. In either case, random effects are unlikely to produce identical topologies for two independent trees with six lineages (*P* = 0.0095). Random effects are also unlikely to explain linkage between nuclear and mitochondrial haplotypes determined in single infections of the same parasite lineages (four cases with mtDNA SYAT01 and five with mtDNA SYAT02, obtained from blackcaps captured in different geographical areas and times, were never observed to interchange nuclear sequences, Fisher exact *P* = 0.0079, Fig. 2). Therefore, we concluded that these parasites remain reproductively isolated, both during evolutionary time and at present. Arguably, such pattern could be due to long-term selfing in isolated populations leading to genetically “clonal” diversification of various strains of the same parasite species [13]. However, two observations argue against this possibility. First, we often observed two or three members of this parasite flock co-infecting individual blackcaps, with 82% of the flock members sometimes co-infecting individual blackcaps together with other flock members, and many apparently reproductively isolated lineages participated in such mixtures (Fig. 4). Recurrent coexistence of parasite lineages in vectors' bloodmeals should therefore bring about frequent interbreeding opportunities [1]. Second, parasite transmission rate was very high, as demonstrated by 92% of young blackcaps becoming infected soon after leaving the nest [14], which should prevent the long-term maintenance of “clonal” lineages resulting from selfing [13]. Note also that *Haemoproteus parabelopolskyi* parasites of blackcaps undergo normal sexual reproduction in the vector [15], and strict clonality resulting from asexual reproduction has never been demonstrated for *Plasmodium* parasites [13]. Given these facts, reproductive isolation between coexisting lineages is the most likely explanation for the observed linkage between parasite mitochondrial and nuclear haplotypes [3], [13], although the isolating mechanisms involved remain to be uncovered.

### Testing for host and parasite population subdivision

Blackcaps were sampled in seven breeding populations distributed between southern Spain and Sweden, but there was no geographic structure in the genetic variation contained in the parasite flock infecting this species (AMOVA: Φ_ST_ = −0.01; d.f. = 6,121; *P* = 0.60, estimated genetic variance among populations = 0). This result supports the hypothesis that the diversification of blackcap malaria parasites occurred without long-term population subdivision.

In addition, blackcap populations show an extremely weak neutral genetic structure across their geographical range [16]. Therefore, we conclude that blackcap parasites are very unlikely to have diverged during historical periods of host population isolation. Supporting this view, various European passerines that show strong evidence of population fragmentation in the past [17], and pairs of sister species which apparently diverged in different glacial refugia [18], did not show the diversity of monophyletic parasites found in blackcaps, even if phylogenetic analyses support a long-term association between these species and their parasites [11], [18].

## Discussion

Our results imply that the blackcap parasite flock consists of sympatric biological species [3], which have diverged within a single avian host species without population subdivision. It is important to note that some of these parasites show clear ecological differences, such as different times of transmission (seasonal or year-round), dispersal rates, or local prevalence [14]. If such diverse life histories mean that these parasites exploit different adaptive optima within the same host species, natural selection for ecological specialization could trigger parasite speciation without host switching [19]. In addition, the fact that parasites undergo sexual reproduction in the vector's gut make us anticipate that intricate associations between blackcap parasites and *Culicoides impunctatus* biting midges (the vector of *Haemoproteus parabelopolskyi* in blackcaps [15]) may play a relevant role as causes of both parasite reproductive isolation and restricted host range of this group of parasites.

Our results are the first to show that sympatric, within-host divergence possibly occurs in malaria parasites. This divergence mode has important implications because diverse but closely related parasites infecting the same host species may establish complex ecological interactions [14], [20]. Such scenarios may intensify disease impacts on populations, because a diverse parasite community can greatly constrain a host's life history strategies and ultimately fitness by imposing a larger antigenic variation for the host's immune system [21], and perhaps by boosting up virulence as a consequence of within-host competition among closely related parasites [22]. Malaria parasites are economically and socially important [1], [23]. Therefore, understanding the origins of diversity in these parasites transcends the interest of evolutionary biologists [9], such knowledge being essential for disease control and wildlife conservation [24].

## Materials and Methods

Blood samples of wild birds were obtained at different European localities from southern Spain to Sweden, at migration stopover sites, and in European and African wintering areas. We screened 4513 wild birds in total, corresponding to 48 passerine species (47 European species plus the African hill babbler; Table 1).

We detected malaria infections by amplification of 479 bases of the parasite cytochrome *b* gene using DNA extracted from bird blood and highly efficient polymerase chain reaction (PCR) methods [25], [26]. Different malaria lineages were distinguished by one or more nucleotide differences [3]. Multiple infections revealed by mixed sequences were resolved by TA-cloning [27]. In total, we scored 1927 infected birds, the average sample size being 40 infected individuals per species (median = 9.5, range 1–357; Table 1). We found 143 distinct parasite lineages, each one being found in 1.9 species on average (range 1–22 species). In 13 blackcap cases (from distantly located sites or different years), we also amplified 220 bases of the nuclear DHFR-TS gene as described previously [3]. All these sequences were retrieved from singly infected blackcaps, so that the association between parasite nuclear and mitochondrial DNA sequences could be unambiguously determined. We could not retrieve the sequence of this gene from all blackcap parasites because many occurred in mixed infections or were not amplified using our PCR [3]. The DNA sequences used in this study have been deposited in GenBank (Table 1).

We used PAUP [28] to construct a maximum likelihood phylogenetic tree based on parasite cytochrome *b* sequences (Fig. 2), using a General Time Reversible model of nucleotide substitution with gamma parameter α = 0.623, and assumed proportion of invariable sites = 0.427. This was the best of 56 models according to the Akaike information criterion implemented in Modeltest [29]. Support to internal branches was estimated by bootstrap analyses (1000 replicates) [28]. We confirmed the tree by repeating the analysis using Bayesian methods as implemented in mrBayes 3.0 [30], under the same model of nucleotide substitution. This method produced the same tree topology, and similar or even stronger support for internal branches, as evaluated by posterior probabilities derived from trees sampled every 500 generations from a 10-million generations Markov Chain Monte Carlo series, with a burn-in time of 250000 generations that removed any trees generated before convergence had been reached. To construct the DHFR-TS tree, we used PAUP and a Kimura 3-parameters model with unequal base frequencies [28], [29]. The exact probability of obtaining identical topologies for trees based on nuclear and mitochondrial genes was calculated by generating all possible trees with six leaves using COMPONENT 2.00a [31].

We analysed the genetic structure of the blackcap parasite flock using an analysis of molecular variance (AMOVA) [32], comparing seven breeding populations covering most of the species' range, from southern Spain to Sweden (Fig. 3). The analysis used Kimura two-parameter distances under a gamma distribution with α = 0.12, as estimated from the data. The significance of the fixation index (Φ_ST_) was tested by 5000 permutations of parasite haplotypes among populations [32].

Aside from blackcaps, we extensively sampled 14 other species (*n*>40 birds and >25 scored infections), which were used to estimate intraspecific parasite richness (Fig. 1, Table 1). To avoid sampling effects, curves of cumulative lineage richness (addition of new parasite lineages as new infected hosts are inspected) were constructed, and the number of parasite lineages found after scoring 25 infections was used as a standard estimate of parasite richness (R_25_). Average curves and R_25_ values (±S.E.) were derived from 1000 richness curves constructed by randomly changing the order in which individual hosts were screened. While the number of parasite lineages found in one species depended on the number of infections scored (*r* ^2^ = 0.58, *n* = 48, *P*<0.0001), R_25_ was independent of sample size (*r* ^2^ = 0.04, *n* = 15, *P* = 0.43).

## References

[1] Kreier JP (1994). Parasitic protozoa. 2nd edition..

[2] Pérez-Tris J, Hasselquist D, Hellgren O, Krizanauskiene A, Waldenström J, Bensch S (2005). What are malaria parasites?. Trends Parasitol.

[3] Bensch S, Pérez-Tris J, Waldenström J, Hellgren O (2004). Linkage between nuclear and mitochondrial DNA sequences in avian malaria parasites – multiple cases of cryptic speciation?. Evolution.

[4] Ricklefs RE, Fallon S, Bermingham E (2004). Evolutionary relationships, cospeciation, and host switching in avian malaria parasites.. Syst Biol.

[5] Waldenström J, Bensch S, Kiboi S, Hasselquist D, Ottosson U (2002). Cross-species infection of blood parasites between resident and migratory songbirds in Africa.. Mol Ecol.

[6] de Meeûs T, Michalakis Y, Renaud F (1998). Santa Rosalia revisited: or why are there so many kinds of parasites in ‘The Garden of Earthly Delights’?. Parasitol Today.

[7] Filchak KE, Roethele JB, Feder JL (2000). Natural selection and sympatric divergence in the apple maggot *Rhagoletis pomonella*.. Nature.

[8] McKoy KD (2003). Sympatric speciation in parasites – what is sympatry?. Trends Parasitol.

[9] Coyne JA, Orr HA (2004). Speciation..

[10] Valkiūnas G, Križanauskienė A, Iezhova TA, Hellgren O, Bensch S (2007). Molecular phylogenetic analysis of circumnuclear hemoproteids (Haemosporida, Haemoprotidae) of Sylviid birds, with description of *Haemoproteus parabelopolskyi*, nov.. J Parasitol..

[11] Hellgren O, Waldenström J, Pérez-Tris J, Szöllősi E, Hasselquist D (2007). Detecting shifts of transmission areas in avian blood parasites - a phylogenetic approach.. Mol Ecol..

[12] Böhning-Gaese K, Schuda MD, Helbig AJ (2003). Weak phylogenetic effects on ecological niches of *Sylvia* warblers.. J Evol Biol.

[13] Razakandrainibe FG, Durand P, Koella JC, de Meeûs T, Rousset F (2005). “Clonal” population structure of the malaria agent *Plasmodium falciparum* in high-infection regions.. Proc Natl Acad Sci USA.

[14] Pérez-Tris J, Bensch S (2005). Dispersal increases local transmission of avian malarial parasites.. Ecol Lett.

[15] Valkiūnas G, Iezova T (2004). The transmission of *Haemoproteus belopolskyi* (Haemosporida: Haemoproteidae) of Blackcap by *Culicoides impunctatus* (Diptera: Ceratopogonidae).. J Parasitol.

[16] Pérez-Tris J, Bensch S, Carbonell R, Helbig AJ, Tellería JL (2004). Historical diversification of migration patterns in a passerine bird.. Evolution.

[17] Bensch S, Hasselquist D (1999). Phylogeographic population structure of great reed warblers: an analysis of mtDNA control region sequences.. Biol J Linn Soc.

[18] Reullier J, Pérez-Tris J, Bensch S, Secondi J (2006). Diversity, distribution and exchange of blood parasites meeting at an avian moving contact zone.. Mol Ecol.

[19] Schluter D (2000). The ecology of adaptive radiation..

[20] Rohani P, Green CJ, Mantilla-Beniers NB, Grenfell BT (2003). Ecological interference between fatal diseases.. Nature.

[21] de Roode JC, Read AF (2003). Evolution and ecology, after the malaria genomes.. Trends Ecol Evol.

[22] de Roode JC, Pansini R, Cheesman SJ, Helinski MEH, Huijben S (2005). Virulence and competitive ability in genetically diverse malaria infections.. Proc Natl Acad Sci USA.

[23] Sachs J, Malaney P (2002). The economic and social burden of malaria.. Nature.

[24] Daszak P, Cunningham AA, Hyatt AD (2000). Emerging infectious diseases of wildlife - threats to biodiversity and human health.. Science.

[25] Waldenström J, Bensch S, Hasselquist D, Östman Ö (2004). A new nested PCR method very efficient in detecting *Plasmodium* and *Haemoproteus* infections from avian blood.. J Parasitol.

[26] Hellgren O, Waldenström J, Bensch S (2004). A new PCR assay for simultaneous studies of *Leucocytozoon* spp., *Plasmodium* spp. and *Haemoproteus* spp. from avian blood.. J Parasitol.

[27] Pérez-Tris J, Bensch S (2005). Diagnosing genetically diverse avian malarial infections using mixed-sequence analysis and TA-cloning.. Parasitology.

[28] Swofford DL (2003). PAUP*. Phylogenetic Analysis Using Parsimony (*and Other Methods). Version 4.0b 10..

[29] Posada D, Crandall KA (1998). Modeltest: testing the model of DNA substitution.. Bioinformatics.

[30] Ronquist F, Huelsenbeck JP (2003). MrBayes 3: Bayesian phylogenetic inference under mixed models.. Bioinformatics.

[31] Page RDM (1993). COMPONENT. Ver 2.00a..

[32] Schneider S, Roessli D, Excoffier L (2000). Arlequin ver. 2000: A software for population genetics data analysis..

